# Local disruptions in non‐rapid eye movement sleep expression are associated with cerebrovascular pathology and worse memory performance in older adults

**DOI:** 10.1002/alz.093871

**Published:** 2025-01-09

**Authors:** Destiny E Berisha, Batool Rizvi, Miranda G. Chappel‐Farley, Abhishek Dave, Novelle J Meza, Negin Sattari, Kitty K. Lui, Ivy Y. Chen, Nicholas J. Tustison, Ariel Neikrug, Ruth M Benca, Michael A. Yassa, Bryce A Mander

**Affiliations:** ^1^ Department of Neurobiology and Behavior, University of California, Irvine, Irvine, CA USA; ^2^ University of California, Irvine, Irvine, CA USA; ^3^ Department of Psychiatry and Human Behavior, University of California, Irvine, Irvine, CA USA; ^4^ SDSU / UC San Diego Joint Doctoral Program in Clinical Psychology, San Diego, CA USA; ^5^ Center for the Neurobiology of Learning and Memory, University of California Irvine, Irvine, CA USA; ^6^ Wake Forest School of Medicine, Wake Forest University, Winston‐Salem, NC USA

## Abstract

**Background:**

Aging is associated with disruptions in non‐rapid eye movement (NREM) sleep and memory decline. Cerebral small vessel disease (CSVD) increases with age and is associated with clinical sleep disturbance, but little is known about its relationship with local expression of NREM sleep. Here, we explore associations between CSVD burden, memory, and local electroencephalography (EEG) measures during NREM sleep in older adults.

**Methods:**

Fifteen cognitively intact older adults (mean age 71.8±6.3 years, 10 female, Apnea Hypopnea Index (AHI) = 7.82±7.95) underwent T2‐weighted fluid‐attenuated inversion recovery magnetic resonance imaging, an in‐lab overnight polysomnography with 128‐channel EEG, and a sleep‐dependent mnemonic discrimination task was administered. Lobar white matter hyperintensity (WMH) volumes were calculated using a validated semi‐automated toolbox. Topographical correlations were run with 5000‐permutation threshold free cluster enhancement.

**Results:**

Older age was associated with lower absolute posterior total (all p<0.016, r>‐0.61) and slow sigma power (all p<0.018, r>‐0.60) and slow wave activity (SWA) (all p<0.017, r>‐0.61) over centro‐posterior EEG derivations. Age was also positively associated with frontal (t = 0.51, p<0.01) and parietal (t = 0.49, p<0.01) but not occiptal (t = 0.33, p = 0.092) or temporal (t = 0.36, p = 0.064) WMH burden. Occipital WMH burden was also not associated with AHI (t = 0.038, p = 0.843). Occipital WMH burden was associated with global reductions in alpha activity (all p<0.043, t>‐0.78), centro‐posterior reductions in SWA (all p<0.043, t>‐0.63) and Delta (all p<0.009, t>‐0.63), frontal reductions in total (all p<0.043, t>‐0.71) and fast sigma power (all p<0.004, t>‐0.57), global reductions in slow sigma power (all p<0.043, t>‐0.71), and central reductions in theta activity (all p<0.042, t>‐0.57). Better lure discrimination for low similarity lures was significantly positively associated with higher relative slow oscillation expression over fronto‐parietal regions, though this did not survive TFCE correction (all p<0.033, r>0.55).

**Conclusions:**

Collectively, these findings indicate that WMH burden in older adults is associated with local disruptions in NREM sleep expression in multiple frequency bands, of which lower frequencies were particularly vulnerable. This in turn may explain weakened memory performance. Future studies should examine the role of NREM sleep expression in cognitive decline associated with CSVD burden.

